# The Response of Microbiota Community to *Streptococcus agalactiae* Infection in Zebrafish Intestine

**DOI:** 10.3389/fmicb.2019.02848

**Published:** 2019-12-06

**Authors:** Qi-Lin Zhang, Hong-Wei Li, Wei Wu, Man Zhang, Jun Guo, Xian-Yu Deng, Feng Wang, Lian-Bing Lin

**Affiliations:** ^1^Faculty of Life Science and Technology, Kunming University of Science and Technology, Kunming, China; ^2^College of Life Sciences, Nanjing Agricultural University, Nanjing, China

**Keywords:** zebrafish, intestinal microbiota, *Streptococcus agalactiae*, PacBio sequencing, full-length 16S rRNA gene

## Abstract

Recently, *Streptococcus agalactiae* has become a major pathogen leading to Streptococcosis. To understand the physiological responses of zebrafish (*Danio rerio*) to *S. agalactiae*, the intestinal microbiota composition of the intestine (12 and 24 h post-infection, hpi, respectively) in zebrafish infected with *S. agalactiae* were investigated. The intestinal bacterial composition was analyzed using PacBio high-throughput full-length 16S rRNA gene sequencing. The most predominant bacteria in the zebrafish intestine were the Fusobacteria phylum and *Sphingomonas* genus. *S. agalactiae* infection affected the composition of partially intestinal microbiota. At the species level, the relative abundance of the pathogenic intestinal bacteria *Aeromonas veronii*, *S. agalactiae*, and *Clostridium tarantellae* significantly increased after *S. agalactiae* infection (*p* < 0.05), while that of the beneficial intestinal bacteria *Bacillus licheniformis*, *Comamonas koreensis*, and *Romboutsia ilealis* significantly decreased (*p* < 0.05), showing that *S. agalactiae* infection aggravates the zebrafish disease through promoting abundance of other intestinal pathogenic bacteria. This study is the first PacBio analyses of the zebrafish intestinal microbiota community under pathogenic infection. Results suggest that the *S. agalactiae* infection alters the intestinal flora structure in zebrafish.

## Introduction

*Streptococcus agalactiae* is a Gram-positive bacterium belonging to group B *Streptococcus*. *S. agalactiae* infection can cause a variety of fish diseases, such as meningitis, sepsis, ascites, and anorexia, leading to extremely high mortality rates, generally greater than 50% (Su et al., 2016). *S. agalactiae* has been reported to infect more than 30 species of fish (Deng and Wang, 2016), including *Arius felis*, *Cynoscion regalis*, and *Pampus argenteus*. Among susceptible species, tilapia (*Oreochromis niloticus*) is reported to be under the greatest threat from *S. agalactiae* infection. *S. agalactiae* infection occurs frequently in the main tilapia production areas in China, with an incidence rate greater than 90%, and a cumulative mortality rate of up to 90% (Li et al., 2009; Xing et al., 2011). In addition, *S. agalactiae* infection is associated with a mortality rates greater than 50% in trout (*Oncorhynchus mykiss*) and causes a mortality rate of 30–50% in various ornamental and economic fishes in Cyprinidae family (Eldar et al., 1995). *S. agalactiae* has a wide range of hosts and a long duration of infection and can cause high mortality rates in fish, leading to a serious threat for the healthy development of the fishery industry.

The intestinal mucosa plays a key role in the body’s first line of defense. Intestinal cells and their immune factors maintain immune homeostasis and resist invasion from exogenous organisms (Nowarski et al., 2017). Studies have shown that the intestine’s ability to resist exogenous organisms depends on its own immune function and the intestinal microbiota structure (Purchiaroni et al., 2013). Previous studies have found that the abundance and composition of the *Salmonella* genus were significantly different in zebrafish (*Danio rerio*) intestines exposed to *Microcystis aeruginosa* compared to control zebrafish (Qian et al., 2019). In another study, the abundances of *Methylmonas*, *Flavobacterium*, and *Phytophthora* genera in the intestinal flora of grass carp (*Ctenopharyngodon idellus*) with enteropathic disease were significantly different to those with from the control group (Tran et al., 2018). Together, this evidence suggests that the structure of the fish microbiota can respond to pathogens infection in terms of microbial abundance and composition.

Most data on intestinal microbiota have been generated by microbial cultivation in a laboratory, but this approach is limited by the cultivability of gut microbes, because the majority of intestinal microbes cannot be cultured (Li W. et al., 2018). In recent years, culture-independent methods have frequently been used to investigate the richness and composition of gut microbes (Li W. et al., 2018). The 16S ribosomal RNA (16S rRNA) gene is universally presented across prokaryotes and has been used widely to assess the richness and composition of microbial communities in animal intestines, and also in phylogenetic analysis as molecular marker. For example, Yang et al. (2017) found evidence that *Aeromonas hydrophila* (Gram-negative bacteria) challenge changed the intestinal microbiota composition of zebrafish intestines by analyzing 16S rRNA gene sequences using an Illumina MiSeq sequencing platform (second-generation sequencing technology). More recently, Pacific Biosciences (PacBio) single-molecule real-time (SMRT) sequencing, a third generation high-throughput sequencing technology, has been increasingly used for intestinal microbiota 16S rRNA gene sequencing; this method has the advantage of long-read sequencing, which can generate an average length of effective reads greater than 8 kb (Li W. et al., 2018). The PacBio SMRT sequencing is a powerful tool for constructing profiles of bacterial taxonomy and measuring abundance using the full-length 16S rRNA gene (Toma et al., 2014). However, the effect of pathogenic organisms on fish intestinal flora has not yet been tested using PacBio sequencing.

Zebrafish are a well-established model for fish immunology studies (Li D. et al., 2018). Zebrafish have been used to establish a disease model for *Salmonella*-infected fish; researchers have found that genes encoding transcription factors, immune cell surface receptors, cytokines, and chemokines presented respond acutely to *Salmonella* infection (Anita et al., 2011). Similarly, zebrafish have also been employed for the analysis of fish immune responses to skin infections with *Citrobacter freundii* (Lü et al., 2012). Zebrafish were infected with *Staphylococcus aureus* to explore the immune regulatory effects of microRNAs (miRNAs) in gills; we identified a set of miRNAs regulating innate immune processes, apoptosis, and defense and antibacterial responses (Zhang et al., 2019). Therefore, many publications have shown that zebrafish are an ideal model for exploring problems related to bacterial infection of fish.

The exploration of fish intestinal microbiota structural responses to important pathogenic bacteria is necessary to understand the role of intestinal microorganisms in responses to bacterial infection. In this study, zebrafish were used as a model organism, and PacBio high-throughput sequencing technology was combined with bioinformatics to analyze changes in zebrafish intestinal microbiota after *S. agalactiae* infection.

## Materials and Methods

### Animals and *S. agalactiae* Challenge

All zebrafish were treated in accordance with the recommendations from the Guide for the Care and Use of Laboratory Animals. The experimental protocol was approved by the Ethical Committee of Researches of Kunming University of Science and Technology. Wild-type (AB strain) adult zebrafish were purchased from the China Zebrafish Resource Center (CZRC)^1^ (mean weight: 0.37 ± 0.1 g, average body length: 3.3 ± 0.3 cm). Experimental healthy zebrafish were acclimatized for approximately five days to empty their digestive system contents using filtered freshwater, prior to their infection with *S. agalactiae* strain SAM 12 belonging to serotype III, sequence type (ST)-17 (Wessels et al., 1991) (from College of Marine Sciences, Qinzhou University, Qinzhou, China). *S. agalactiae* used in this study was grown and cultured according to methods of Patterson et al. (2012). Notably, Patterson et al. used 10^6^ cfu/mL *S. agalactiae* as the highest concentration in their experiments, and found the intraperitoneal route of infection caused the induction of host inflammatory immune response in the adult zebrafish brain (Patterson et al., 2012). Furthermore, a ∼50% survival rate was reported for zebrafish at 24 h post-injection (hpi), indicating that a 10^6^ cfu/mL concentration of *S. agalactiae* can successfully induce the immune response in the whole body of adult zebrafish before 24 hpi (Patterson et al., 2012). Thus, intraperitoneal challenge (IP) of 10^6^ cfu/mL and 24 h were considered optimal for an acute immune response for use in downstream investigations. Eighty zebrafish individuals were maintained in two aquariums (40 zebrafish per 60 L aquarium). For the treatment group, 10 μL (1 × 10^6^ cfu/mL) of *S. agalactiae* in PBS buffer was injected into each of the 40 zebrafish enterocoelia following our previous method (Zhang et al., 2019). For the control group (named as Drgc), the same volume of filtered PBS buffer was injected without *S. agalactiae*. Slight anesthesia using 0.02% tricaine (Sigma–Aldrich, United States) before injection was used to avoid excessive fish activity. The clinical signs (e.g., hemorrhage of fin ray base, exophthalmos, and gill hyperemia) of treatment groups of 12 and 24 hpi were presented in Supplementary File 1.

### Zebrafish Intestine Sampling

At 12 (named as Drgt12) and 24 hpi (Drgt24), ten adult zebrafish individuals were randomly collected from each of the Drgt12 and the Drgt24 groups, placed on ice for cold anesthesia, and then the zebrafish intestine was collected using a scalpel and pair of forceps. The zebrafish intestine from ten individuals that were collected at each time point was then pooled into the Drgt12 and the Drgt24 samples, respectively. Notably, due to focusing on population-level changes of microbiomes in this study, so pooling microbiome samples prior to DNA amplification and metagenomics sequencing to estimate community-level diversity was a viable, as reported in methods of Ray et al. (2019). The zebrafish intestine collected from these two treatment groups was placed in a 2 mL sterile RNase-free centrifuge tube. Sample collection was also conducted in parallel for the control groups. The experimental procedure was performed two times independently to give two biological replicates (control, 12, and 24 hpi). All samples were collected and stored at −80°C until use.

### DNA Extraction, PCR Amplification and Library Preparation

Genomic DNA was extracted from samples using a TIANamp Stool DNA Kit (TIANGEN Biotech, China), according to the manufacturer’s instructions. The purity and concentration of DNA were detected using agarose gel electrophoresis, and further assessed using a NanoDrop 1000 spectrophotometer (Thermo Scientific, United States).

Purified DNA was diluted to 1 ng/μL, with a total of 40–50 ng, for each sample. Diluted genomic DNA as a template, was used to amplify the full-length 16S rRNA gene sequence using Phusion^®^ High-Fidelity PCR Master Mix with GC Buffer (NEB, United Kingdom) containing specific primers with six barcode (V1–V9, forward primer: AGRGTTTGATYMTGGCTCAG, reverse primer: GGYTACCTTGTTACGACTT) and high-efficiency high-fidelity enzymes (NEB, United Kingdom). The amplicon was detected following 2% agarose gel electrophoresis, and purified using a QIAquick Gel extraction kit (Qiagen, Germany). The purified PCR products were used to construct sequencing libraries using a SMRT Bell Template Prep Kit (Pacific Biosciences, United States). In brief, the sequencing adapters were ligated to both ends of the PCR products using DNA-binding enzyme, and the DNA fragment was further purified using AMpure PB magnetic beads. Next, the purified fragment was buffer-resolved, then a BluePippin system was used to screen the target fragments with specific sizes, and DNA fragments were further purified using AMpure PB magnetic beads. The concentration of the constructed library was determined using a Qubit 3.0 Fluorometer (Invitrogen, United States), and size of insert fragments was confirmed using an Agilent 2100 chromatography (Agilent, United States).

### PacBio Sequencing and Analysis of the Full-Length Bacterial 16S rRNA Gene

The libraries were sequenced using a PacBio RS II DNA Sequencing System (Pacific Biosciences, United States). The data from each sample were distinguished according to barcode sequences. The protocol RS_ReadsOfInsrt, available in SMRT Portal (version 2.7, PacBio), was used to correct the raw data with the following parameters: minimum predicted accuracy of 90%, sequences with a length <1,340 or >1,640 bp were removed. Subsequently, cutadapt 2.3 software (Martin, 2011) was used to remove primers on sequences, and chimeric DNA sequences were detected using the UCHIME algorithm (Edgar et al., 2011). Chimeric sequences were discarded according to previous studies (Haas et al., 2011) to obtain clean data. Clean reads from each sample were clustered using UPARSE software (v7.0.1001) (Edgar, 2013), namely sequences in each sample with 97% identity were classified as operational taxonomic units (OTU). The most frequently occurring sequence in an OTU was selected as the representative of OTUs for further analysis. Species annotation analysis was conducted using Mothur software (v.1.36) (Schloss et al., 2009) by searching (threshold of 0.8∼1) species taxonomy information in the SSUrRNA database of SILVA (v128) (Quast et al., 2013). Taxonomic information of bacterial populations in each sample was calculated at following levels: kingdom, phylum, class, order, family, genus, and species. Beta and Alpha diversity indexes (e.g., Observed-otus, Chao, Shannon, Simpson, Ace, Good’s-coverage) were calculated in the Quantitative Insights In to Microbial Ecology (QIIME) package (version. 1.9.1) (Caporaso et al., 2010).

### Statistical Analyses

Intergroup differences in intestinal flora were analyzed by one-way ANOVA plus Bonferroni post-tests in IBM SPSS Statistics 2. Results are presented as the mean ± standard deviation (SD). Results with *p*-values <0.05 were considered to be statistically significant.

## Results and Discussion

### Statistical Analysis of Sequences

**FIGURE 1:**
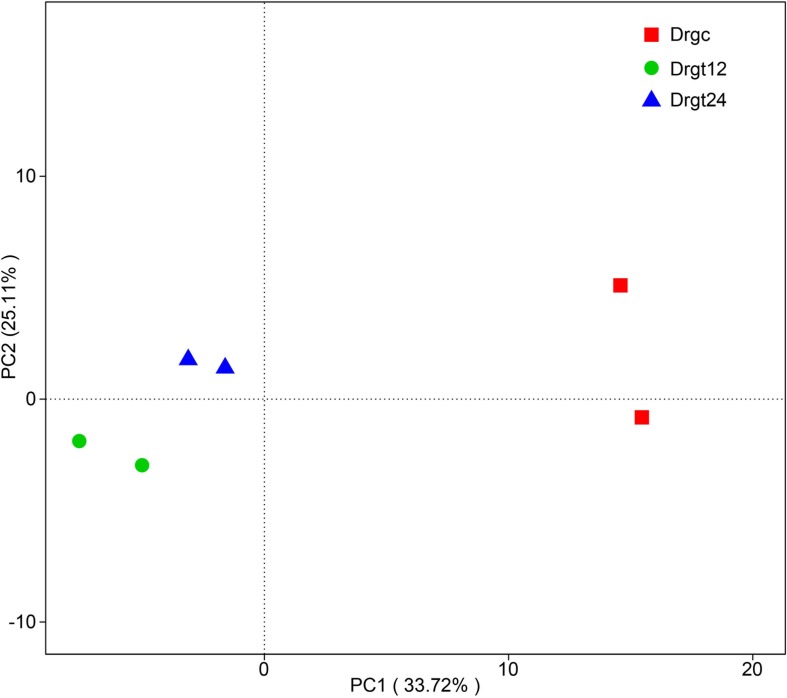
Principal component analysis (PCA) of bacterial community composition from six samples. Drgc indicates control groups; Drgt12 and Drgt24 indicate treated groups collected at 12 and 24 hpi, respectively.

A total of 78,724 clean reads were obtained from six samples, each sample with ∼1,449–1,471 bp average length (Supplementary File 2). By comparing average length of 16S rRNA gene sequences to those from previous studies of intestinal microbiota communities analyzed by Illumina sequencing technology (∼349–400 bp) (Zheng et al., 2019), there is an obvious advantage in terms of the read lengths generated by PacBio. A total of 362 OUT types were obtained for the six samples (which is similar to previous reports at a level of 97% for adult zebrafish (Udayangani et al., 2017). Based on beta diversity index calculated by UniFrac distance of OUT among all the six samples, the similarity of bacterial community structure in zebrafish intestine samples was compared. PCA results show that two samples from 12 hpi are concentrated on the left side with those from 24 hpi, while two samples from the control group were obviously separated from those from treatment groups (Figure 1), indicating that significant differences exist between control and treated samples. Moreover, two samples belonging to each group presented as a close cluster in comparison with other samples, indicating that effective biological replicates were obtained in this study.

Based on annotated OTUs, an average of 106, 52, and 47 bacterial species were detected in control, 12 and 24 hpi treated samples, respectively (Table 1), suggesting that *S. agalactiae* infection can significantly decrease the number of bacterial species among the intestinal microbiota of the zebrafish intestine. Furthermore, the estimators of community richness (ACE and Chao), diversity (Shannon and Simpson) and coverage (Good’s coverage) are presented in Table 1. Results from the analysis of alpha diversity metrics show that the microbial richness and diversity decreased significantly after *S. agalactiae* infection (*p* < 0.05), but estimators of alpha diversity showed no significant difference (*p* > 0.05) between 12 and 24 hpi. *S. agalactiae* infection thus significantly decreased the community richness and diversity of intestinal bacteria. We speculate that the promotion or maintenance of richness and diversity within intestinal microbiota can enhance the antibacterial ability of zebrafish.

**TABLE 1:** Alpha diversity indexes for each group.

	**Drgc (control)**	**Drgt12 (12 hpi treated groups)**	**Drgt24 (24 hpi treated groups)**
Observed species	106 ± 17^a^	52 ± 11^b^	47 ± 6^b^
Shannon	2.49 ± 0.38^a^	1.12 ± 0.11^b^	0.78 ± 0.10^b^
Simpson	0.54 ± 0.09^a^	0.32 ± 0.04^b^	0.21 ± 0.04^b^
Chao	150.31 ± 16.16^a^	27.92 ± 1.83^b^	27.17 ± 5.54^b^
Ace	148.13 ± 13.02^a^	30.66 ± 2.76^b^	32.23 ± 5.29^b^
Good’s coverage	0.99	0.99	0.99

*Different superscripts identify significant differences (*p* < 0.05).*

### Analysis of Microbial Community Composition at the Phylum Level

**FIGURE 2:**
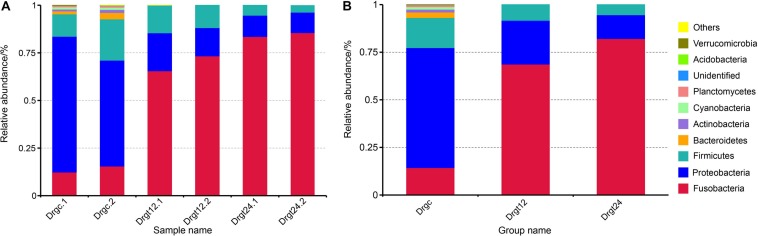
The top 10 phylum relative abundance (%) in the intestinal bacteria from all samples. The results are presented in **(A)** each sample, and in **(B)** each group. Drgt12 and Drgt24 indicate treated groups collected at 12 and 24 hpi, respectively. Number after decimal point indicates biological replicates.

A total of 12 phyla were detected in all six zebrafish intestine samples, the top nine of which was presented in samples and groups (Figures 2A,B), including the Fusobacteria, Proteobacteria, Firmicutes, Bacteroidetes, Actinobacteria, Cyanobacteria, Planctemycetes, Acidobacteria, and Verrucomicrobia. Among these, the phyla, Fusobacteria, Proteobacteria, Firmicutes, were dominant both in control and treatment groups, with a significant difference between control and the two treatment groups, respectively (*p* < 0.05). Firmicutes and Bacteroidetes were reported to be the most dominant phyla in mammals (Udayangani et al., 2017), and Fusobacteria and Proteobacteria in fish (Huang et al., 2018; Zhang et al., 2019). Fusobacteria were previously found to be the highest proportion of phylum within the intestinal flora of adult zebrafish (Bates et al., 2006; Wong et al., 2015). In the present investigation, the intestinal flora diversity at the phylum level in the intestine of control and *S. agalactiae* infected zebrafish were characterized, and results were similar to previous studies. Furthermore, in this study, the relative abundance of Fusobacteria was 13.76 ± 3.2% in the control group, 68.82 ± 5.93% in the 12 hpi group, and 79.14 ± 1.25% in 24 hpi group. Proteobacteria and Firmicutes were 63.74 ± 10.12 and 17.94 ± 6.76%, respectively, in the control group, 22.71 ± 4.97 and 8.18 ± 2.62%, respectively, in the 12 hpi group, and 13.12 ± 1.77 and 4.26 ± 1.33%, respectively, in the 24 hpi group. The relative abundance of Fusobacteria was significantly greater in the infected group than the control group (*p* < 0.05), while that of Proteobacteria and Firmicutes was significantly less in the infected groups compared to the control group. However, these primary phyla showed no significant difference (*p* > 0.05) between 12 and 24 hpi. This observation shows that *S. agalactiae* infection primarily influenced the intestinal microbiota abundance rather than the composition type at a bacterial phylum level. In addition, a high good’s coverage (0.99) suggests that the PacBio sequencing adequately covers intestinal bacteria, which is better than that from the second generation high throughput sequencing (e.g., Illumina sequencing) (∼0.93) (Zheng et al., 2019). Long-read sequencing thus shows some advantages in the sequence coverage of fish intestinal microbiota.

### Analysis of Microbial Community Composition at Genus and Species Level

Fifty-two genera were detected in all the three groups, of which 44 bacterial genera were detected in the control group, and 39 and 41 bacteria genera in the 12 and 24 hpi groups, respectively. *Sphingomonas*, *Cetobacterium*, *Bacillus*, *Aeromonas*, *Streptococcus*, *Ureibacillus*, *Plesiomonas*, *Enterococcus*, *Bradyrhizobium*, and *Acetobacter* were the main genera (the sum of the relative abundance >1%) in all the three groups (Table 2). *Sphingomonas*, *Cetobacterium*, *Bacillus*, *Aeromonas*, and *Streptococcus* were the dominant genera, whose relative abundance was >10% of all in the three groups. This result was in agreement with previous results from Stephens et al. (2016) regarding the microbiota of zebrafish across development, and results regarding the intestinal microbiota of zebrafish fed chitosan-silver nanocomposites (Udayangani et al., 2017). In the present study, among genera, six (*Sphingomonas*, *Cetobacterium*, *Aeromonas*, *Streptococcus*, *Plesiomonas*, *Acetobacter*) showed a significant difference (*p* < 0.05) between the control group and at least one of the treatment groups. This study revealed that *S. agalactiae* infection altered relative abundance patterns of genera within the zebrafish intestinal microbiota. In addition, most of these genera showed no significant difference (*p* > 0.05) between 12 and 24 hpi, but two genera (*Acetobacter* and *Streptococcus*) showed a significant difference (*p* < 0.05), exhibiting their change dynamics of the relative abundance in *S. agalactiae* disease process.

**TABLE 2:** The top 10 genera and species relative abundance (%) of intestinal bacteria from all samples from control and treatment zebrafish groups.

**Taxonomic categories**		**Drgc (%)**	**Drgt12 (%)**	**Drgt24 (%)**
Genus	*Sphingomonas*	66.84 ± 5.67^a^	31.10 ± 2.82^b^	34.01 ± 1.74^b^
	*Cetobacterium*	1.33 ± 0.17^a^	32.53 ± 3.97^b^	34.65 ± 2.96^b^
	*Bacillus*	11.57 ± 1.98	8.01 ± 0.99	7.01 ± 2.33
	*Aeromonas*	0.01^a^	6.41 ± 1.43^b^	4.95 ± 1.87^b^
	*Streptococcus*	0.08 ± 0.02^a^	6.90 ± 1.17^b^	3.18 ± 0.12^c^
	*Ureibacillus*	2.58 ± 0.39	1.95 ± 0.42	2.62 ± 0.46
	*Plesiomonas*	0.01^a^	3.36 ± 0.92^b^	2.65 ± 0.96^b^
	*Enterococcus*	2.04 ± 0.49	1.94 ± 0.17	1.77 ± 0.40
	*Bradyrhizobium*	1.17 ± 0.09	1.57 ± 0.38	1.63 ± 0.13
	*Acetobacter*	1.18 ± 0.10^a^	1.22 ± 0.21^a^	0.49 ± 0.13^b^
	Unidentified	2.36 ± 0.28	1.77 ± 0.63	2.13 ± 0.58
	Other	10.8 ± 2.27	6.62 ± 1.19	7.94 ± 2.06
Species	*Bacillus licheniformis*	8.35 ± 1.53^a^	2.17 ± 0.72^b^	1.26 ± 0.22^b^
	*Aeromonas veronii*	0^a^	4.93 ± 1.65^b^	3.78 ± 0.64^b^
	*Achromobacter marplatensis*	2.34 ± 0.32	2.42 ± 0.67	2.75 ± 0.71
	*Streptococcus agalactiae*	0^a^	4.57 ± 1.25^b^	2.56 ± 0.77^c^
	*Enterococcus faecium*	1.94 ± 0.57	1.49 ± 0.40	1.67 ± 0.44
	*Bacillus thermoamylovorans*	1.62 ± 0.31	1.73 ± 0.39	1.25 ± 0.33
	*Bradyrhizobium elkanii*	1.15 ± 0.19	0.91 ± 0.31	0.88 ± 0.17
	*Clostridium tarantellae*	0.03^a^	0.26 ± 0.06^b^	0.14 ± 0.03^c^
	*Comamonas koreensis*	0.21 ± 0.06^a^	0.02^b^	0.01^b^
	*Romboutsia ilealis*	0.23 ± 0.10^a^	0.01^b^	0.02^b^
	Unidentified	5.74 ± 1.14	2.73 ± 0.69	3.21 ± 1.02
	Others	78.39 ± 4.19	79.77 ± 5.61	84.58 ± 4.76

*Different superscripts identify significant differences (*p* < 0.05) of relative abundance. Average values without standard deviation (SD) means the SD values <0.01%.*

Research is yet to fully explore the species of the fish intestinal microbiota; many publications have focused only on genera or higher taxonomic ranks. Based on results from this study, the bacterial species with the top 10 relative abundance totaled by all the groups are listed in Table 2. Six species (*Bacillus licheniformis*, *Aeromonas veronii*, *S. agalactiae*, *Clostridium tarantellae*, *Comamonas koreensis*, and *Romboutsia ilealis*) were significantly different (*p* < 0.05) between the control group and at least one treatment group, which may be caused by their respective changes of ecological niches resulting from *S. agalactiae* invasion. Previous studies have shown that *B. licheniformis* could increase digestive enzyme activity and growth in the fish *Allogynogenetic crucian* (Liu et al., 2005). *C. koreensis* is widely distributed in soil and can reduce Fe^3+^/HS to contribute to organic biodegradation, which can be used as a probiotic supplement (Wang et al., 2010). *R. ilealis* is a natural inhabitant and a key player in the small intestine of mammals (Gerritsen et al., 2017). In this study, these three beneficial bacteria had significantly (*p* < 0.05) less relative abundance in the control group compared to treatment groups, indicating that pathogenic microorganism of fish, such as *S. agalactiae* in this study, can induce a increasing beneficial bacteria. This may be explained by that *S. agalactiae* invasion stimulated host intestine to create favor conditions for growth of several beneficial bacteria. Conversely, *A. veronii* is a pathogenic bacterium that can cause disease in humans and fish (Rahman et al., 2002). The roles of *C. tarantellae* in fish disease remain largely unknown, but *C. botulinum* has been frequently reported to be pathogenic in fish and contamination of fish or sea products (Cann et al., 1965; Novotny et al., 2012). The relative abundance of these three harmful bacteria was significantly greater (*p* < 0.05) in *S. agalactiae* infected groups compared to the control group. A large number of evidence showed that normal immune activity of organisms strongly depended on the interaction between immune system and intestinal flora, such as pathogen elimination and probiotic tolerance of the immune system (Luo et al., 2014). For example, toll-like receptors (TLRs) and microbial associated molecular patterns (MAMPs) from the intestinal flora were considered to play an key role in discrimination between pathogens and probiotics by host immune system, because TLRs recognition of MAMPs can activate innate and adaptive immune responses (Luo et al., 2014). However, results from current study overall suggest that *S. agalactiae* infection facilitates the growth of pathogenic bacteria but inhibits the growth of intestinal probiotics in zebrafish. This probably attributed to immune function deactivation caused by *S. agalactiae* invasion, and then ecological niches of probiotics were occupied by the increased pathogenic population. Notably, the relative abundance of two species of pathogenic bacteria (*S. agalactiae* and *C. tarantellae*) showed significantly decreased, indicating that immune system or unfavorable factors in zebrafish intestine probably hampered proliferation of several pathogens in disease process. However, to deeply understand their roles in *S. agalactiae* disease progression, dynamic changes of their relative abundance need to be further revealed through increasing more sampling time points after zebrafish infection.

Intraperitoneal injection of *S. agalactiae* was associated with the detection of *S. agalactiae* in the zebrafish intestine of *S. agalactiae* groups, and this observation was further confirmed using PCR for control and treated groups of adult individuals (Supplementary File 3). Moreover, previous studies provided morphologic evidence that *S. agalactiae* seems to possess filamentous structures that interact with the microvilli of the enterocytes in tilapia (*Oreochromis* sp.) (Vásquez-Machado et al., 2019). Therefore, *S. agalactiae* colonization of the intestinal mucosa should be a future focal point for the prevention and cure of fish diseases caused by *S. agalactiae*. Notably, it was shown that the bacterial capsule impairs the attachment of *S. agalactiae* to intestinal epithelium of tilapia, and an acidic environment could favor the adhesion of encapsulated strains (Barato et al., 2016). These findings provided an idea for a successful anti-adherence therapy to prevent streptococcosis in fish.

## Conclusion

This study shows the first high-throughput analysis of the intestinal microbiota structure in fish after bacterial infection using PacBio full-length 16S rRNA gene sequencing technology. The major composition (community richness and diversity) of intestinal bacteria in zebrafish was significantly affected by *S. agalactiae* invasion, while it did not showed significant difference between two stages (12 and 24 hpi) of *S. agalactiae* infection. Fusobacteria, Proteobacteria and Firmicutes were the dominant phyla in the zebrafish intestinal microbiota, and *Sphingomonas*, *Cetobacterium*, and *Bacillus* were the most abundant genera. *B. licheniformis*, *A. veronii*, *A. marplatensis* had the greatest relative abundance at the species level. This study provides a theoretical basis for the intestinal microecology of fish, and the development of intestinal microbial resources for the prevention and control of fish *S. agalactiae*-derived disease.

## Data Availability Statement

The datasets generated for this study can be found and downloaded in Figshare public repository, https://doi.org/10.6084/m9.figshare.c.4723157.v1.

## Ethics Statement

The animal experiments in the present study were approved by the Animal Ethics Committee of Kunming University of Science and Technology.

## Author Contributions

L-BL and Q-LZ conceived and designed the study. Q-LZ, WW, and H-WL performed the experiments. Q-LZ, WW, MZ, JG, and X-YD analyzed the data. H-WL, MZ, and FW performed the statistics. Q-LZ, H-WL, and WW drafted the manuscript. L-BL and WW revised the manuscript. All authors have read, commented on, and approved the manuscript.

## Conflict of Interest

The authors declare that the research was conducted in the absence of any commercial or financial relationships that could be construed as a potential conflict of interest.

## Footnotes

1 http://en.zfish.cn/
