# Supplementary material for: The Response of Microbiota Community to Streptococcus agalactiae Infection in Zebrafish Intestine
Source: Front Microbiol. 2019 Dec 6;10:2848. doi: 10.3389/fmicb.2019.02848 (PMC6908962; doi:10.3389/fmicb.2019.02848)
Supplement: Supplementary file 1 [file Presentation_1.PDF]

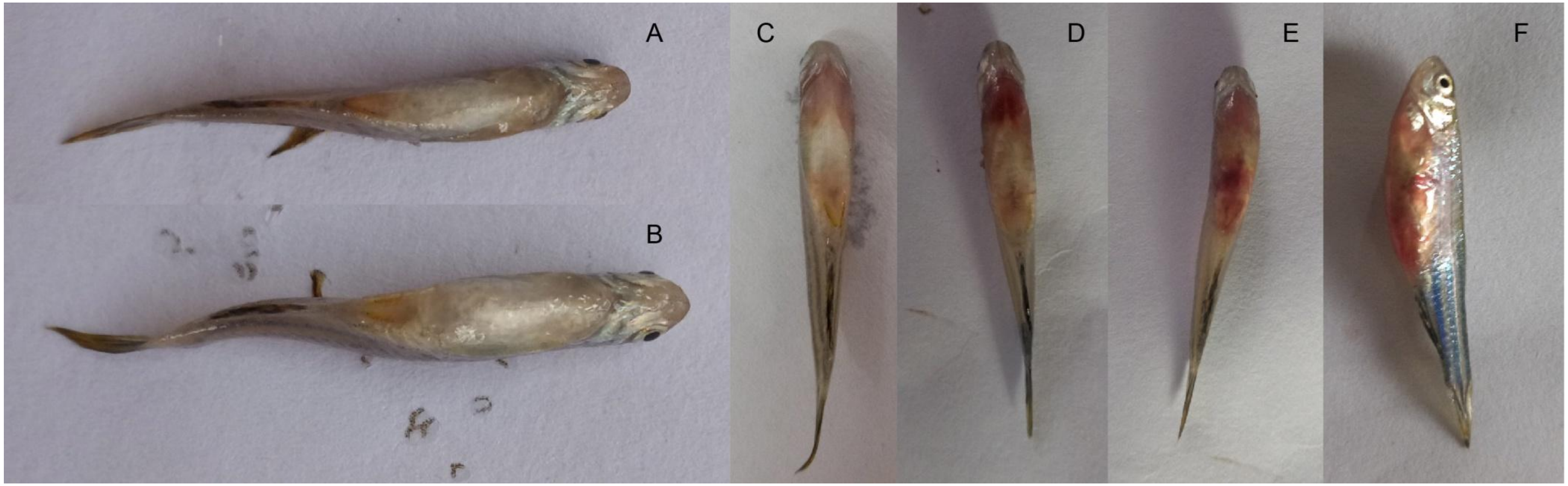

Figure S1 Disease signs of adult zebrafish infected by *S. agalactiae* and control. A-B, control individuals injected by PBS at 24 hpi; C-D, individuals infected by *S. agalactiae* at 12 hpi; E-F, individuals infected by *S. agalactiae* at 24 hpi.
