# Supplementary material for: The Response of Microbiota Community to Streptococcus agalactiae Infection in Zebrafish Intestine
Source: Front Microbiol. 2019 Dec 6;10:2848. doi: 10.3389/fmicb.2019.02848 (PMC6908962; doi:10.3389/fmicb.2019.02848)
Supplement: Supplementary file 2 [file Presentation_2.PDF]

Supplementary File 2 Statistics of sequencing data from each sample.

Sample name	Raw reads	Clean reads	Average length (nt)	Effective (%)	OUT types
Drgc.1	12288	12112	1454	98.57	136
Drgc.2	13080	12873	1459	98.42	124
Drgt12.1	13586	13431	1449	98.86	92
Drgt12.2	12729	12475	1452	98.01	89
Drgt24.1	13671	13322	1450	97.45	86
Drgt24.2	14814	14511	1471	97.95	93
