# Supplementary material for: The Response of Microbiota Community to Streptococcus agalactiae Infection in Zebrafish Intestine
Source: Front Microbiol. 2019 Dec 6;10:2848. doi: 10.3389/fmicb.2019.02848 (PMC6908962; doi:10.3389/fmicb.2019.02848)
Supplement: Supplementary file 3 [file Presentation_3.PDF]

Experimental process: To validate none of *S. agalactiae* in zebrafish intestine in control groups, the specific primers (F: CCACACTGGGACTGAGACAC; R: TCGGGTGAACAACTCTCGTG) were used for PCR analysis from a sample of the same DNA that was used for the PacBio sequencing. Each PCR reaction was performed in a total volume of 25 μL, containing 2.5 μL of 10x PCR reaction buffer (with $Mg2+$), 1.5 μL of dNTPs (each 2.5 mM), 1.5 μL of each of two primers (20 μM), 2 μL of the extracted DNA, 0.2 μL Taq DNA polymerase (5 U/μL, TAKARA, Japan), and 16.8 μL of distilled water. The PCR conditions were as follows: 95 ℃ for 5 min, 35 cycles of 95 ℃ for 30 s, a primer-specific annealing temperature of 55 ℃ for 30 s, 72 ℃ for 1 min, and a final extension at 72 ℃ for 10 min. All PCR products were separated by electrophoresis on a 1.0% agarose gel.

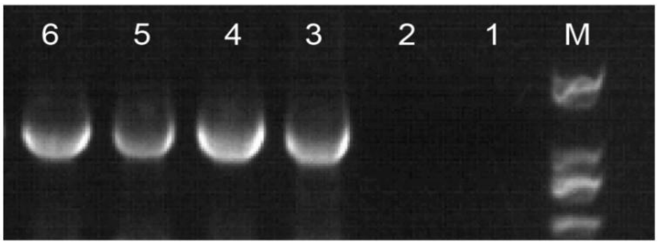

Figure S2 16S amplicon of *S. agalactiae* in each sample. M: DL2000 DNA marker (Takara, Japan); 1-2 indicated Drgc.1 and Drgc.2, respectively; 3-4 indicated Drgt12.1 and Drgt12.2, respectively; 5-6 indicated Drgt24.1 and Drgt24.2, respectively.
